# Assessment of the accuracy of 3D printed medical models through reverse engineering

**DOI:** 10.1016/j.heliyon.2024.e31829

**Published:** 2024-05-24

**Authors:** Yosef Wakjira, Navaneethan S. Kurukkal, Hirpa G. Lemu

**Affiliations:** University of Stavanger, Faculty of Science and Technology, Department of Mechanical and Structural Engineering and Materials Science, Kjell Arholms Gate 41, 4021, Stavanger, Norway

**Keywords:** Accuracy, Deviation, Medical model, Reverse engineering, 3D scanning

## Abstract

The dimensional accuracy of additively manufactured (3D printed) medical models can be affected by various parameters. Although different methods are used to evaluate the accuracy of additively manufactured models, this study focused on the investigation of the dimensional accuracy of the medical model based the combination of reverse engineering (RE) and additive manufacturing (AM) technologies. Human femur bone was constructed from CT images and manufactured, using Fortus 450mc Industrial material extrusion 3D Printer. The additive manufactured femur bone was subsequently 3D scanned using three distinct non-contact 3D scanners. MeshLab was used for mesh analysis, while VX Elements was used for post-processing of the point cloud. A combination of the VX Inspect environment and MeshLab was used to evaluate the scanning performance. The deviation of the 3D scanned 3D models from the reference mesh was determined using relative metrics and absolute measurements. The scanners reported deviations ranging from −0.375 mm to 0.388 mm, resulting in a total range of approximately 0.763 mm with average root mean square (RMS) deviation of 0.22 mm. The results indicate that the additively manufactured model, as measured by 3D scanning, has a mean deviation with an average range of approximately 0.46 mm and an average mean value of around 0.16 mm.

## 1 Introduction

Additive manufacturing (AM) or 3D printing is a technology that is currently being used in several application areas, as adding materials layer by layer has given the manufacturing industry a plethora of choices. AM technologies are widely used in aerospace [1], automotive [2], construction [3], supply chain [4], food [5], electronics [6], medical [7], repairing structures [8], energy [9] and sustainability and recycling [10]. They have been used in various engineering applications from rapid prototyping to low-volume manufacturing to highly customized businesses. Similarly, the procedure of gathering design information is crucial in industries that demand high levels of customization. Consequently, reverse engineering (RE) is another technique that complements AM. RE is the technique of analyzing products to obtain their design information [11,12] for use in many fields, including software, defense systems, aerospace, medical, automotive, consumer electronics, sports equipment, toys, and jewelry [13]. The process of RE involves assessment and analysis for reinvention of a product, in contrast to the design processes focusing on creativity and originality.

The medical field is one that has seen extensive use of both AM and RE. Customized medical equipment can be produced with 3D printing, which is typically not possible with traditional manufacturing techniques. Design information's for such objectives is typically obtained through medical imaging like computed Tomography (CT), magnetic imaging resonance (MRI) or 3D scanning, which is essentially reverse engineering. For example, the human body can also be considered a system through which components (such as body parts and organs) can be reexamined and researched in situations when there is a shortage of "design data". Reverse engineering can serve as a valuable instrument for understanding the human body.

One of primary issue in additive manufacturing for medical is the accuracy of model geometry. The additive manufacturing machine, AM process, and material utilized can affect medical model accuracy. An effective method for planning complex surgeries, for instance, is to create precise medical models from images like digital imaging and communication (DICOM) files [14,15]. Several studies have explored additive-manufactured medical models' dimensional inaccuracies. Chamo et al. [16] assessed the accuracy of patient-specific implants used in craniofacial surgery, using computer-aided design (CAD) AM technology. Salmi et al. [17] studied the accuracy of selective laser sintering (SLS) and polyjet technologies, through a coordinate measuring machine. Likewise, much of the literature has reported the inaccuracies of anatomical models used in the surgical process, for teaching (education) purposes, and in implants printed using different additive manufacturing technologies [[18], [19], [20]].

The aim of this study is to conduct an investigation into the impact of reverse engineering on the evaluation of the accuracy of medical models, through a literature study and experimental work. The results of the deviation and inaccuracy from different 3D scanning technologies are compared.

## 2 Background on RE for accuracy

Reverse engineering with 3D scanning has been used in various fields, including medicine, to assess surgical guide precision. The RE approach was utilized by Giordano et al. [21] to evaluate dental implant surgical guides' accuracy. It is proved to be an effective mechanism for assessing accuracy and ensuring quality control. Works by Russo et al. [22] and Liang et al. [23] mention further uses of RE to assess accuracy. Individualized medicinal supplies are one of RE's many medical applications. Patients can now have customized implants and prostheses. According to Noor et al. [24], a personalized implant for bone fracture created from RE process reduces tension and mobility among the implant and damaged bone. Additional to the purpose of design and production, RE has also been used for the verification purpose. Kloesel et al. [25] used the RE procedure to properly implant a medical device for patient, also used for hand orthotics [26] and soles [27].

Medical reverse engineered model accuracy assessment approaches include.

• medical image acquisition,
• medical image conversion technique,
• model AM technology, and
• deviation measurement method and analysis.

The summary of image acquisition, model construction, accuracy assessment, and deviation analysis of various previous research can be seen on Table 1.

**Table 1:** Methods employed for accuracy assessments.

Image Acquisition	DICOM to STL	AM (3DP) Technology	Method of Deviation Measurement	Software for Deviation Analysis	Reference
**CT**	OsiriX	FDM	Electronic caliper	N/A	[18]
**CT**	Materialise OsiriX	SLM	Non-contact and contact 3D scanning	GOM Inspect	[28]
**CT**	OsiriX	FDM	Digital calipers & Physical measurements	–	[29]
**CT**	3D Slicer	–	CT	Meshlab	[30]
**CT/MRI**	Mimics	VP	Calipers and Micrometers	–	[31]
**CT**	Synapse3D	FDM	CT Scan	–	[32]
**Cone Beam CT**	3D Slicer STL Model Creator	ColorJet Printing (CJP)	3D Scan	GOM Inspect	[33]

Table 2 summarizes previous works in which studies of the accuracy of additive manufactured (3D printed) models using different conditions are reported, various techniques for evaluating the accuracy of the medical models through 3D scanning and RE are presented in Table 3.

**Table 2:** Research articles investigating the accuracy of 3D printing (3DP) medical models.

Objective	Method	Results	Conclusion	Reference
Evaluate accuracy of industrial SLS printer with Fused Deposition Modeling (FDM) printers	Both printers used 100, 250, and 500-μm layers to 3D print CT scans of dry skulls.	In terms of model accuracy, SLS was somewhat better than FDM (0.44 %, 0.52 %, 1.1 % variations).	It has been found that consumer-grade FDM models are accurate enough for planning maxillofacial surgery.	[18]
Analyze the effects of automatic and manual thresholding	Using both manual and automatic thresholding, get images of the head's bone structure from cadaver heads and STL models.	The STL models produced by manual thresholding outperform those of default thresholding.	Pattern recognition and machine learning-based innovation	[19]
Compare referee model errors to models developed with different CT methods and thresholding parameters. Errors in end-implant AM	Using a pig's head, models with different thresholds and CT scans are printed. Error detected by CAD software. To identify printing errors, the implant was 3D printed	Bone structure and printed implant were assessed for AM faults using CT scanning, 3D modeling, and other technologies.	Precision tools are needed to create patient-specific anatomic geometries. Focusing on each phase ensures quality control and prevents treatment or restoration	[28]
Assess the accuracy of AM-generated models of the femoral head	Cadaveric femur CT scans. Digital vernier calipers measuring 3D printed femur models and organic bone	The 3D-printed femur model has a deviation of −0.22 mm–0.099 mm.	3D printed medicsl models can be used for preoperative planning and other medical applications.	[29]
Evaluate the accuracy of three-dimensional liver models	3D models of the liver created from computed tomography scans. Afterward, dimensional analysis is performed by CT scanning the printed models.	The deviation of 1.92 mm was significantly higher than the others. Contrarily, the height of the CT slices was greater than the median deviation.	The effects of 3DP medical model-based pre-operative planning need more investigation.	[30]
Evaluate the accuracy of AM'ed 3D models in identifying spatial relationships and pre-surgical planning across various pathologies	7 medical models 3D printed using different type of resins. The models compared to their original references (CT, MRI and DICOM)	Deviation of < 1 mm dimensional error for all models showed a	Additive manufactured models may be used for presurgical planning and other clinical purposes with a 1-mm inaccuracy	[31]
To evaluate the accuracy surgical models for robotic partial nephrectomy	Comparison of CT scans of related medical models with scans of previously printed models	14 of the 16 scanned models were valid	Anatomically precise printed models aid robot-assisted partial nephrectomy. Assess the 3DP model's surgical assist reliability	[32]
Demonstrate the inaccuracies due to DICOM to STL conversion	Skull files in STL format from three institutes. 3DP models scanned with CAD for dimensional accuracy analysis.	Significant differences exist between the printed model and the initial DICOM	Develop methods for accurately translating DICOM data to CAD/CAM.	[33]
To compare reference model's errors to the model's errors caused by changing parameters.	3D printed model used as a base model and varying model building parameters and 3D print for comparison	Standard model building overestimates the models size. Large curvature areas shows greatest errors	Modification of the standard process of building, in particular the algorithm of segmentation	[34]

**Table 3:** Different methods 3D scanning and RE application for assessment of accuracy of medical models.

Goal	3D Scanning	Mesh Processing so ftware	Mesh Inspection software	Reference
Evaluate the accuracy of printed medical models	Non-contact scanner CT	Geomagic studio MeshLab	Geomagic	[35]
Assess the accuracy of digital impressions from intraoral scanners	Intraoral scanners Non-contact scanners	Geomagic studio	GOM Inspect	[36]
Assess intraoral scanning accuracy to evaluate alignment	Intraoral scanner CT scanning	N/A	GOM Inspect	[37]
Compare dental cast surface curvature and scanning accuracy	Industrial non-contact 3D scanners and intraoral 3D scanners	N/A	GOM Inspect	[38]
Evaluate measuring techniques of AM-created mandible model	Non-contact scanners	Geomagic	GOM Inspect	[39]
Estimate 3D printed mandible model accuracy	Non-contact scanners CMM		GOM Professional	[40]
Building database of ear for use in biometric applications	Non-contact scanner	VX Elements	MeshLab	[41]
Medical model creation as digital resources in a virtual environment	Non-contact scanner	MeshLab	CloudCompare	[42]

## 3 Materials and methods

### 3.1 3D model segmentation

A 40-year-old male (weighing 80 kg) human femur bone's DICOM files from CT scan images were obtained from the Stavanger University Hospital (SUS). Then the DICOM files were converted to. stl (stereolithography) file after segmentation in MIMICS 24.01 software. The process involves thresholding, region growth and masking. The.stl file of the femur bone 3D model file was processed through 3-Matic and exported for additive manufacturing using a material extrusion machine.

### 3.2 Post-processing

**Fig. 1:**
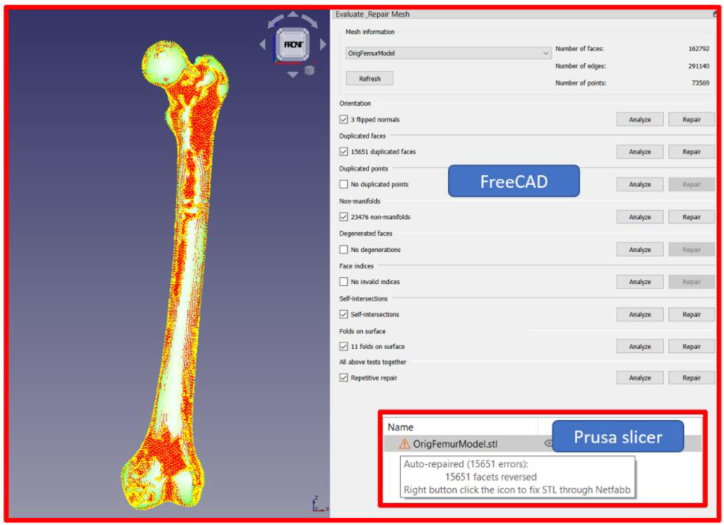
Mesh analysis in FreeCAD and Prusaslicer.

As illustrated in Fig. 1, the STL file that was exported from Mimics contained an errors. After importing the model into FreeCAD, the Prusaslicer was utilized to show up any potential slicing software issues. The Prusaslicer error message indicates that various errors, including incorrect slicing, will occur in the slicing process if it is not processed. MeshLab was used for the mesh file's post processing and cleaning.

### 3.3 Model additive manufacturing

**Figs. 2:**
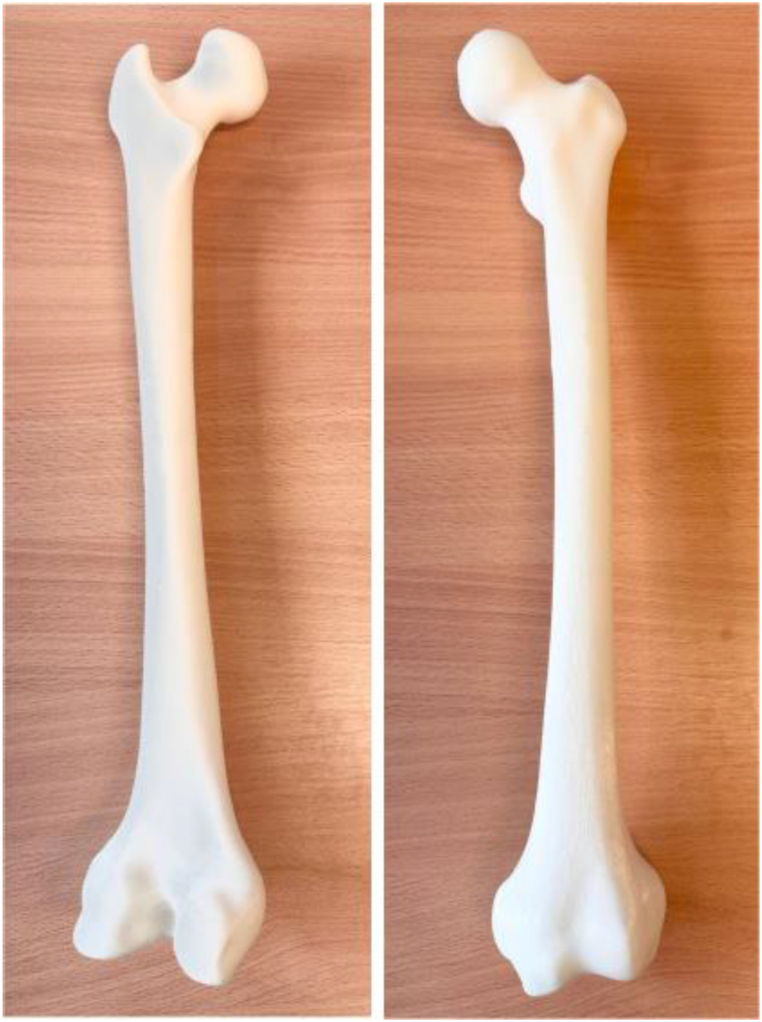
3D Printed model of femur bone and its printing parameters.

The femur model as seen on Fig. 2 was printed using FDM 3D printer, the Fortus 450mc. The STL file generated using 3 Matic software was subsequently transferred to the slicing program, Insight, which serves as the control center of the printing machine and guides the exact location and orientation for printing. Table 4 provides the 3D printing parameter used to print the femur model.

**Tables 4:** 3D printing parameter.

Printing Parameter	Value
Printing nozzle size	0.254 mm (T16) for model 0.178 mm (T12) for support
Nozzle temperature	315 °C for model 293 °C for support
Bed temperature	90 °C
Shell thickness	4 mm
Layer height	0.2542 mm
Total printing time	11.25 h
Number of layers	330

### 3.4 Femur bone 3D scanning

**Fig. 3:**
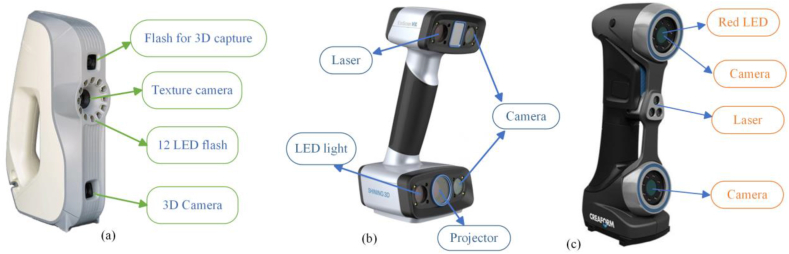
(a) Artec EVA-M, (b) Einscan HX and, (c) Handyscan 700.

The initial stage of reverse engineering physical things is to perform a 3D scan of the component. The bone model, which had been produced by 3D printing, underwent scanning utilizing three handheld non-contact 3D scanners: the Artec EVA-M, Einscan HX, and Handyscan 700, shown in Fig. 3 (a), (b) and (c), respectively. All three scanners are handheld, except that Artec EVA-M featured a portable power supply. Each scanning process's preparation was similar but varied depending on the scanning environment (physical locations) in which the scanners used.

The complete additively manufactured femur model underwent two scans in different orientations before being combined through the post-processing functionalities provided by each 3D Scanners. Selected technical specifications of the 3D Scanners are given in Table 5.

**Table 5:** Manufacturer data for selected hand-held 3D scanning systems.

	Artec EVA-M	Einscan HX	Handyscan 700
**Scanning method**	Structured light	Structured light	SP (Triangulation and binocular vision)
**Acquisition speed (pts/s)**	18000000	1200000	480000
**Light source**	Flashbulb + 12 Led array	LED	7 laser crosses and an additional line
**Range (m)**	0.4–1	0.3–1	0.3
**Accuracy (mm)**	0.1	0.05	0.03
**Resolution (mm)**	0.2	0.25–0.3	0.05
**Scanning area (mm)**	370 x 270	420 x 440	275 x 250
**Depth of field (mm)**	200	200	250
**Data processing**	Geometry and texture based	Markers/features/hybrid/texture	Markers
**Accompanying software**	Artec Studio	Shining3D	VX Elements
**Weight (kg)**	0.9	0.71	0.85

3D Scanners and scanning procedure.

**Fig. 4:**
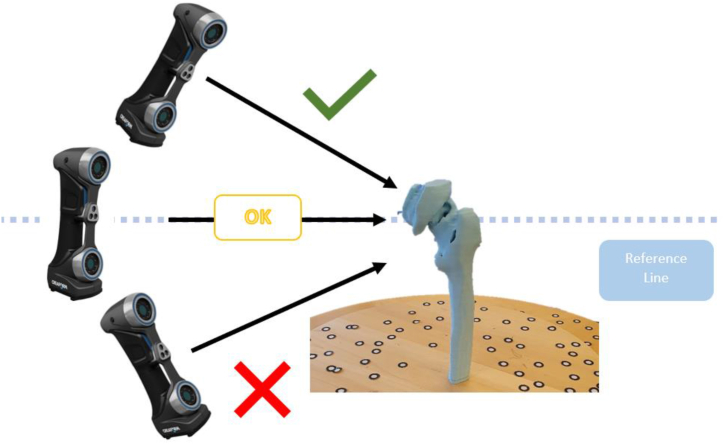
A suitable 3D scanning angle for a femur model.

Varying scanning angles can lead to either strong coherence or substantial deviation. In order to mitigate the potential impact of scanning angle variations and ensure that the scanning cameras capture all necessary information, trial tests were conducted from various angles to determine the ideal angle and position for the model. The angle used for scanning may be shown in Fig. 4, as determined from the trial test.

**Fig. 5:**
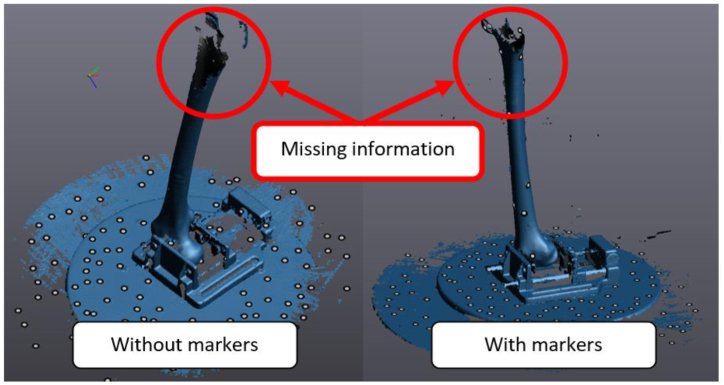
Illustration of missing information when scanned with and without markers scanned by Handyscan 700 3D Scanner.

Despite the use of retroreflective markers, as shown in Fig. 5 (right), scanning the whole femur on the revolving platform proved unsuccessful. The scanning objects may be too skinny to have enough surface area to triangulate, or there may not be enough markers. The Handyscan 700 (Fig. 3 (c)) reliance on retroreflective markings for tiny items makes it problematic. Fig. 5 shows the missing information recorded on the Handyscan 700 3D scanner when scanning with and without markers.

**Figs. 6:**
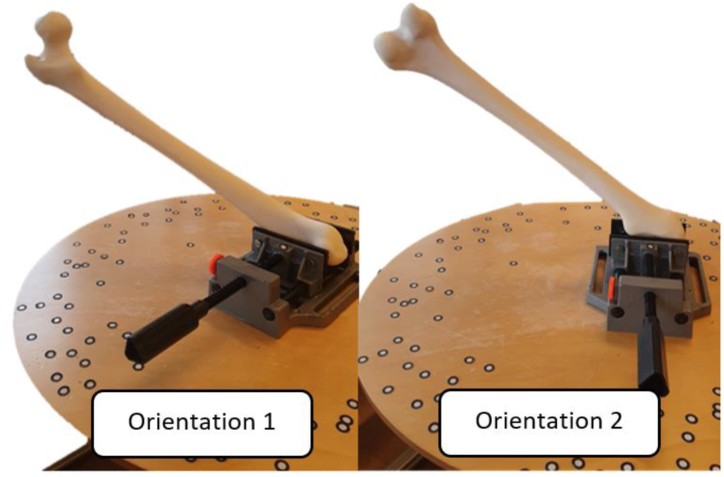
3D Printed model setup in two different orientations of femur for scanning.

This was resolved by placing the femur model to optimize marker and platform capture while minimizing scanning height. Fig. 6 shows the orientations used for 3D scanning.

**Fig. 7:**
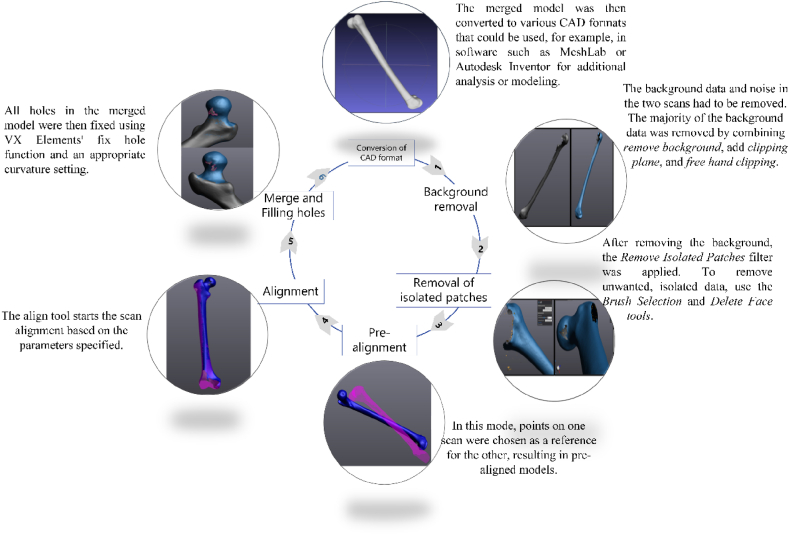
Femur scan post-processing in VX Elements (Handyscan 700).

Scanning took minutes and was well set up. VX Scan was utilized for both orientations on the same project for post-processing. The scanned data was cleaned, fixed, and aligned and overall post process procedure is shown in Fig. 7. VX Scan offers a means of merging and aligning scans. Factors for the alignment can be.

• Target best fit: aligns two scans by imposing a tolerance and a minimum number of matching points
• Surface best fit – aligns images by maximizing the space between them
• Global registration - Three or more scan images are aligned and merged.

The surface best-fit option appears appropriate for aligning femur images because the model's midsection is the same in both scans. This program was then used to align the two scans and combine them into one model.

**Fig. 8:**
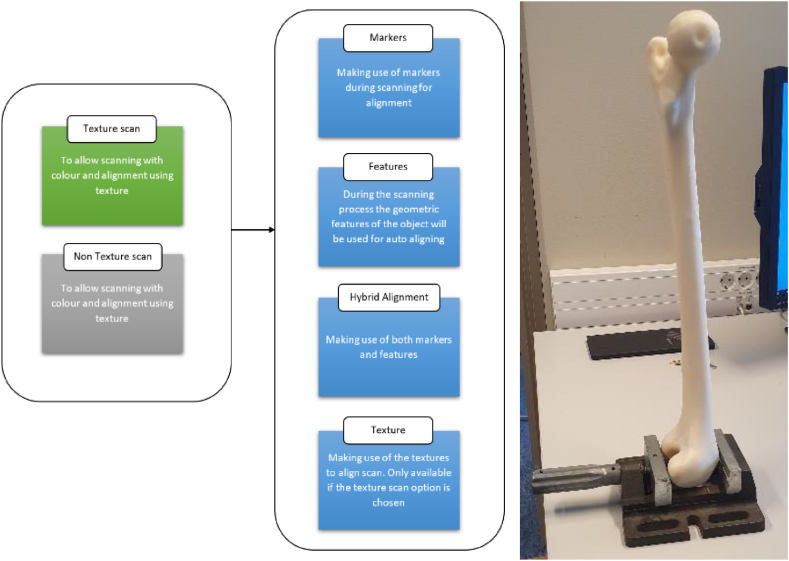
Shining3D pre-setting and model setup for scanning using Einscan HX.

The Einscan HX (Fig. 3 (b)) can act as a portable or stationary 3D data scanner. With Shining3D software, the scanner offers many alignment pre-settings. Fig. 8 (right) shows the femur model being set up on a vice for scanning using Einscan HX. The object was 3D scanned using feature-based, non-texture scan method due to the femur has an organic geometry.

**Fig. 9:**
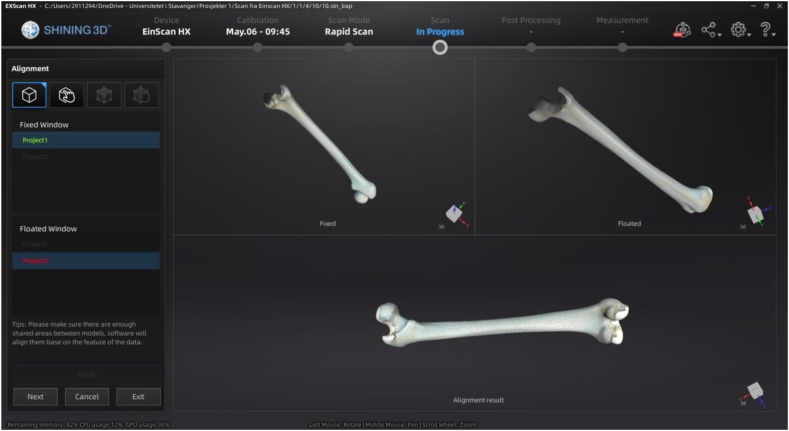
Shining3D femur scans alignment.

The phases in Shining3D's post-processing workflow, as shown in Fig. 7, include eliminating unnecessary data and patches, aligning and integrating the two scans, and removing background information. The scanned file using Shining3D was around 3 Mb in size and saved in ASC format. Fig. 8 displays Shining3D presetting and model setup of the femur on a vice for scanning. Also, Fig. 9 shows the alignment of femur scans in Shining3D.

**Fig. 10:**
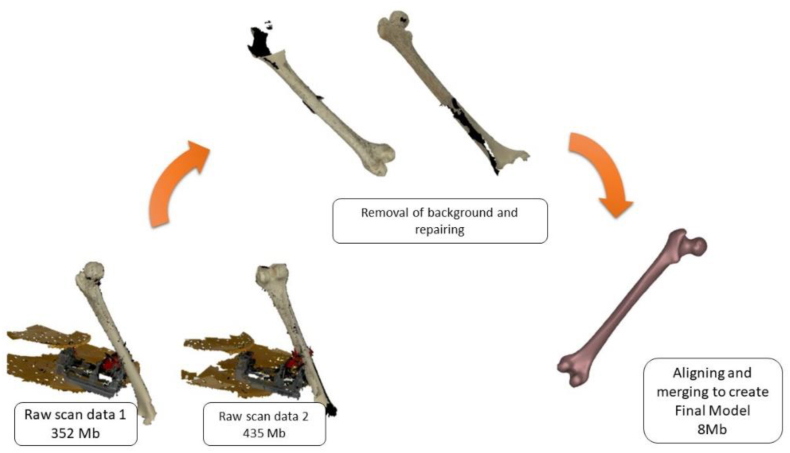
Scanning process using Artec EVA-M.

In contrast to other 3D scanners, the Artec EVA-M (Fig. 3(a)) has a portable power source and scans with less difficulty. The Artec Studio program includes numerous scanning and alignment options, featuring a high-definition scanning option that use its own algorithms to ensure little impact from post-processing. The post-processing for Artec EVA-M also follows the procedure outlined in Fig. 7. Fig. 10 depicts the model transformation summary to create the final 3D model from the raw scanned model.

### 3.5 Deviation analysis

The deviation analysis presented in this paper focuses solely on the discrepancies arising from the 3D printing and scanning procedures. The study does not evaluate the anatomical correctness of the underlying anatomical structure, but rather uses the variance of the models from the STL file generated by the image reconstruction to measure accuracy.

Since the original femur model could not be aligned with any of the three STL models, each scan was examined independently. After importing each model into MeshLab and VX Inspect, the initial femur model served as a reference. The purpose of VX Inspect was to evaluate the accuracy of the models and find out how they differed from the original STL model by comparing the models generated by the three scanning techniques. Using surface best fit, the 3D scanned models from each scanner were matched with the reference model. Color maps, which give tolerance-based deviations, are used to show model differences from the reference model. Tolerance for these scanned models was set at −0.5 mm–0.5 mm. During the analysis, many data points were collected from diverse perspectives and cross-sections.

**Fig. 11:**
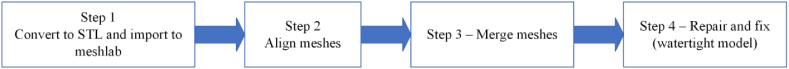
Post processing steps in MeshLab.

In MeshLab, the first step for deviation analysis is to conduct the aligning process. Large files generated by raw data from each scanned entity typically necessitate substantial processing capacity for processing, manipulation, and handling. One alternate approach to file simplification is to do post-processing operations, such as course segmentation, mesh alignment, and post-processing. Fig. 11 below lays out the four primary stages of the suggested process for meshing scans in MeshLab.

In step 1, once the conversion of each scan to STL file format is completed, it will be imported to MeshLab one at a time. The files imported use different local and global coordinate systems and are not in a plane-aligned relationship. It is necessary to align the two scans to integrate them. The iterated closest point (ICP) technique is widely recognized as one of the finest algorithms for aligning point clouds or meshes. By utilizing a transformation matrix incorporating rotation and translation, the technique minimizes the average squared distance between the two-point clouds.

**Fig. 12:**
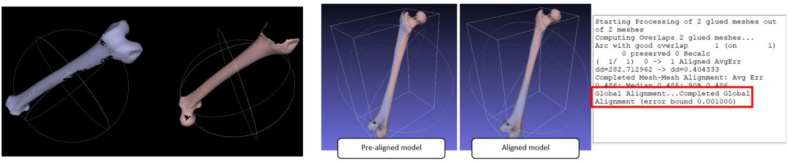
Pre-alignment and completed global alignment.

**Fig. 13:**
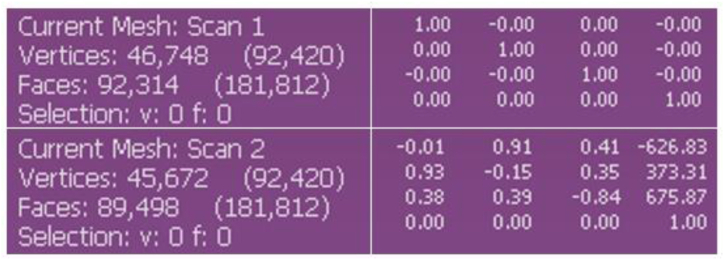
Differences between reference and transformed transformation matrices.

Next, the two scans are aligned using MeshLab aligning tool, which is built on the ICP algorithm. When the dialogue box for alignment opens, freeze one of the scans to serve as a reference model. The scan with a higher number of vertices was merged using point-based gluing. The pre-alignment process involves selecting a minimum of four common locations on each scan model to align the scans. Then the models aligned. The aligned models together with the error bound can be seen on Fig. 12. To confirm the transformation, as illustrated in Fig. 13, we compare the transformation matrices of scans one and two.

**Fig. 14:**
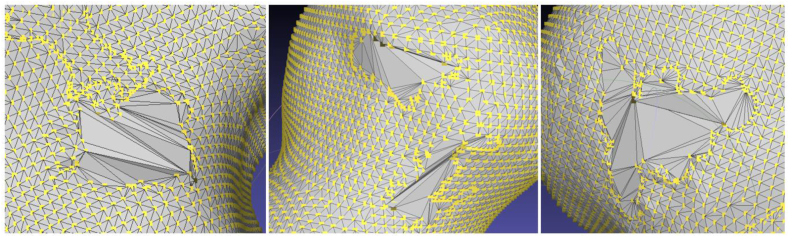
Holes fixed using close function in MeshLab.

Step 3 involves aligning the meshes, and then applying the flat visible layers function to merge them. Finally, the repair, evaluations, and fix procedure are conducted on the merged model. The elimination of self-intersections, non-manifolds, isolated patches, duplicate faces, and isolated faces is the most prevalent operation in these stages. Maximizing the threshold values allows the close function to close model holes. However, the hole closing was not smooth as seen on Fig. 14, therefore it is recommended to use a default or smaller threshold for smaller holes.

**Fig. 15:**
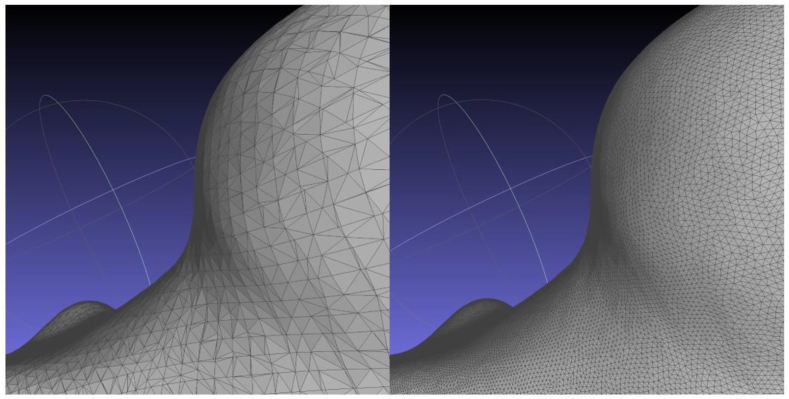
Pre-and post-application of the remeshing filter on the Poisson reconstructed model.

Another potential issue is that the model could not be completely watertight. One practical approach is to make use of MeshLab's screened poisson surface reconstruction function, which is built on the algorithm introduced by Kazhdan et al. [43]. This approach has a major drawback in that it requires a somewhat coarse starting mesh. A proper remeshing tool and smoothing can help solve this issue, Isotropic Explicit Remeshing tool [44], and the Humphrey's Classes (HC) Laplacian Smoothing tool [45] were used. Fig. 15 displays the result of using these filters differently.

After aligning them, several filters incorporated into the model to evaluate the deviation. Filter Distance from reference mesh computes positive and negative model distances. Distance is added as a vertex using this filter. Factors such as deviation distance and vertex data are stored at each data point. A non-binary PLY file containing this data can be exported from MeshLab. We evaluated the models of all the scanners using this metric. The models of all the scanners were evaluated using this metric.

MeshLab also has a Hausdorff distance filter that estimates the absolute distance among the original and scanned femur models, to evaluate the deviation of the printed model from the original CAD model. A calculation of the Hausdorff distance, as described by Shonkwiler [46], forms the basis of the algorithm. The mathematical equation of Hausdorff distance:

$$h\left(A,B\right)=\begin{array}{c} \max\\ a\in A \end{array}\;\left\{\begin{array}{c} \min\\ b\in B \end{array}\left\{d\left(a,b\right)\right\}\right\}$$

Following the alignment of the models in the MeshLab environment, the Hausdorff distance filter was applied. Finally, the information acquired from MeshLab was imported into Matlab in order to produce the box and whisker graphs.

## 4 Discussion of results

### 4.1 Qualitative analysis

**Fig. 16:**
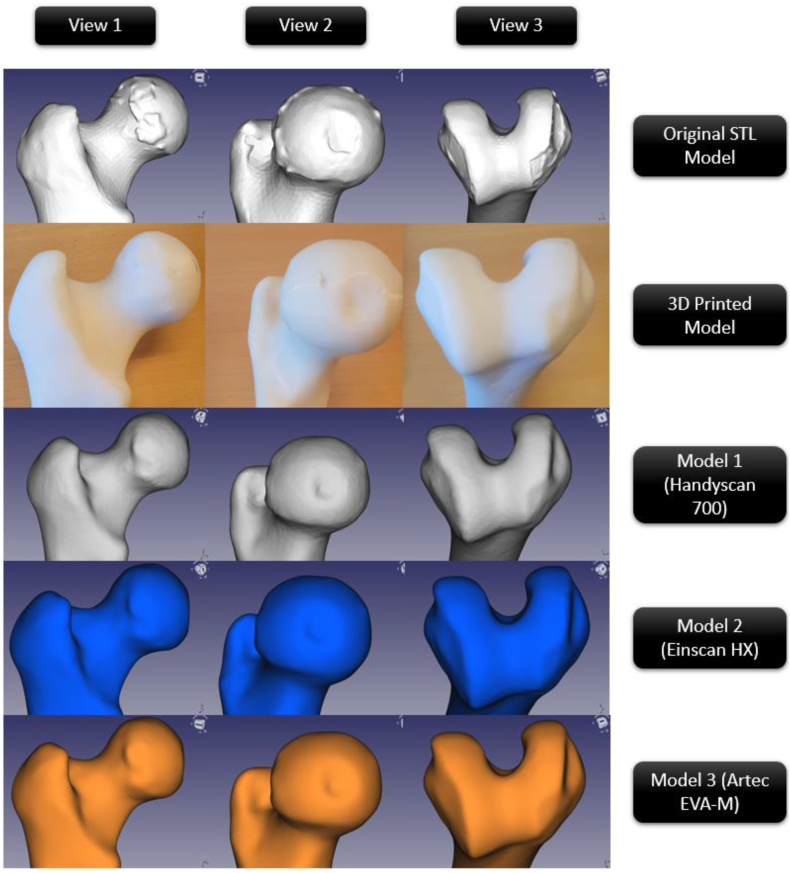
A comparison of the original CAD, 3D printed, and CAD models from three scanning techniques.

The feature-based comparability of the original CAD model, 3D printed model, and CAD models derived from the various scanning procedures is displayed in Fig. 16. Visual examination and image comparison allow for the following deductions to be made.

• The smoothing of all rough edges is the most noticeable change between the original CAD model and the 3D printed model. This is most likely the result of the 3D printer's resolution not being able to reproduce the curvature precisely.
• 3D printing process smoothed any protruding elements on views 2 and 3 (Fig. 16). Since slicing interpolates CAD model section points, this is likely due to the slicing Due to interpolation, this information is lost if the points obtained are too high.
• Views 1, 2, and 3 show that all of the scanners have captured on the unique characteristics of the printed model.

### 4.2 Quantitative analysis

**Fig. 17:**
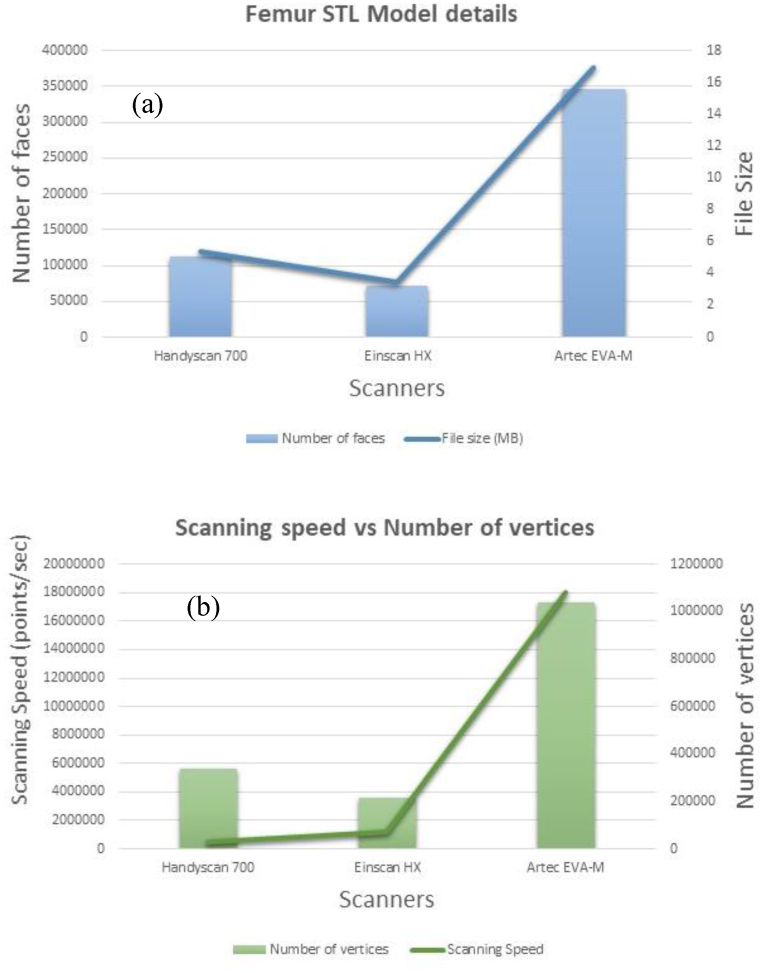
Details of femur STL model (a) comparison of scanning points and (b) comparison of number of vertices.

Each scanner's software examined point clouds from each scan. Models were exported to STL (and others) after post-processing. Fig. 17 shows the clear relation between face number and file size for different scanner models. Fig. 17 (a) shows a comparison of scanning points and (b) shows comparison of number of vertices. Artec EVA-M STL has a higher number of vertices since it can scan 18 million points per second at a high speed.

#### 4.2.1 Deviation analysis results

**Fig. 18:**
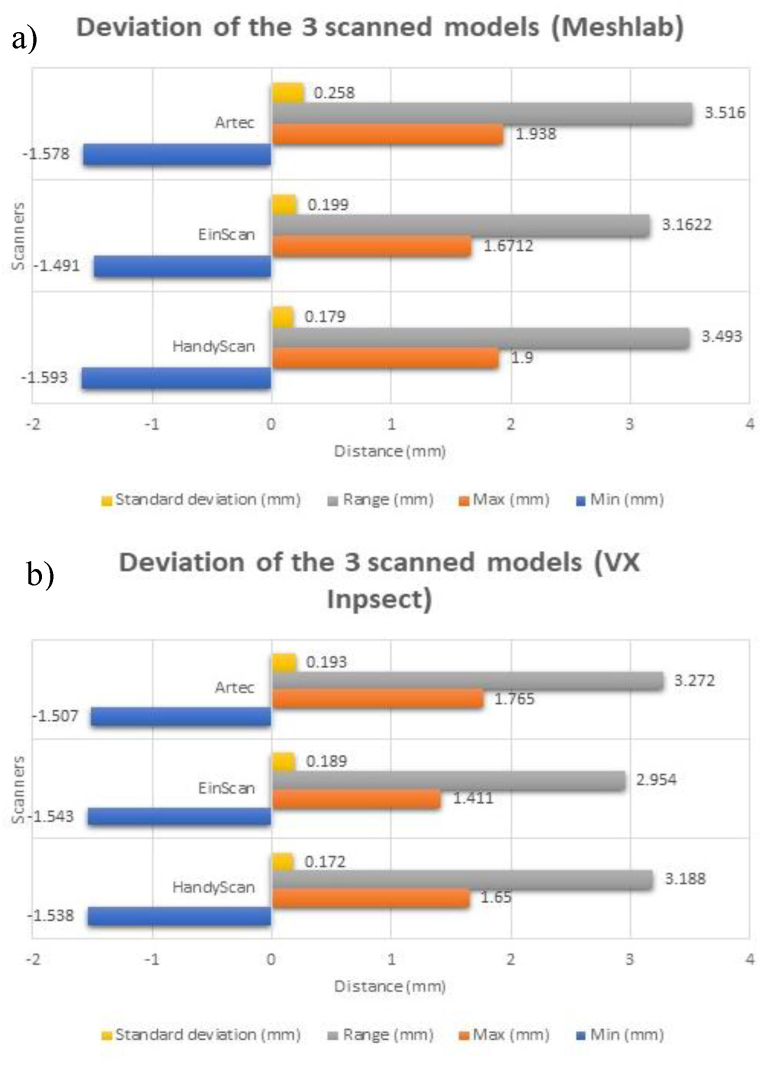
The scanned models maximum, minimum, and standard deviation for (a) Meshlab and (b) VX Inspect.

The color map tool was utilized to identify the discrepancies between the scanned and reference models after the models were aligned in VX Inspect. Each model has this implemented. Fig. 18 displays the statistical data from the software's deviation analysis. The scanned models maximum, minimum, and standard deviation.

Filtering based on distance from reference mesh was implemented following MeshLab model alignment.

**Fig. 19:**
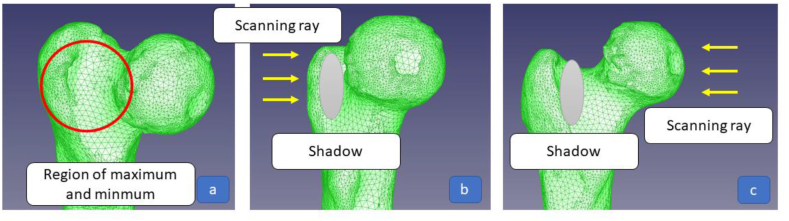
(a) Regions with minimum and maximum deviation, (b) & (c) direction of shadows.

The average absolute minimum and maximum deviations were, respectively, −1.54 mm and 1.72 mm. The average range of deviations are approximately 3.34 mm. Fig. 19(a) shows that the maximum and lowest values of the 3DP model were close (same region on the 3DP model). These discrepancies can be a result of the scanning procedures. Fig. 19(b) and (c) show how the shadows of the femur impact the highlighted region. The region comprises a curved incursion characteristic in the original STL file. A lot of locations in that area were hard to scan because of the shadows. The original curvature is lost when the points are interpolated, making the deviation larger.

#### 4.2.2 Analysis of VX inspect results

**Fig. 20:**
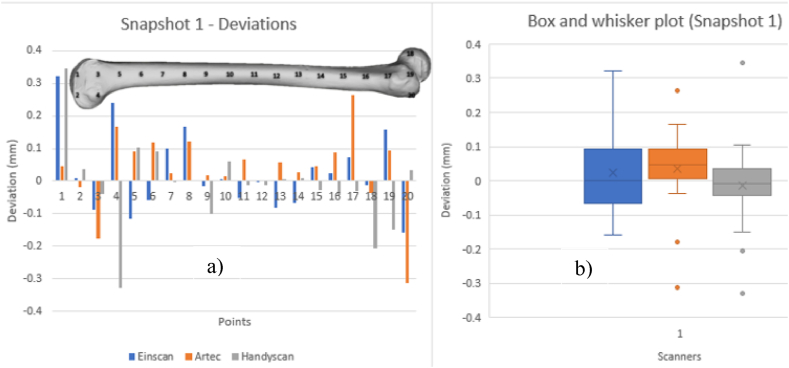
Deviations of scanners (a) and Box and whisker plot for Snapshot 1 (b).

**Fig. 21:**
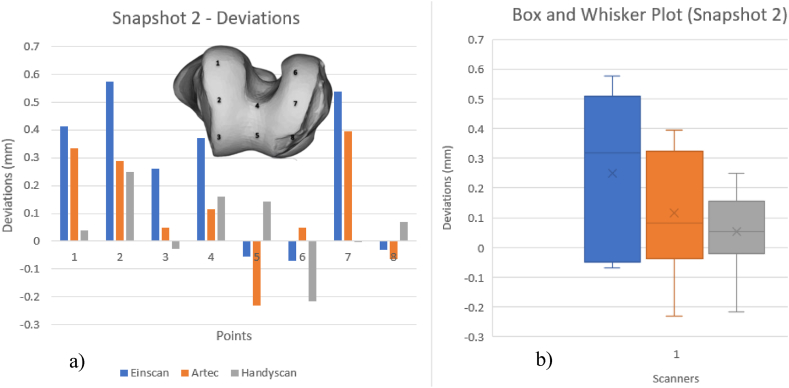
Deviations of scanners (a) and Box and whisker plot for Snapshot 2 (b).

**Fig. 22:**
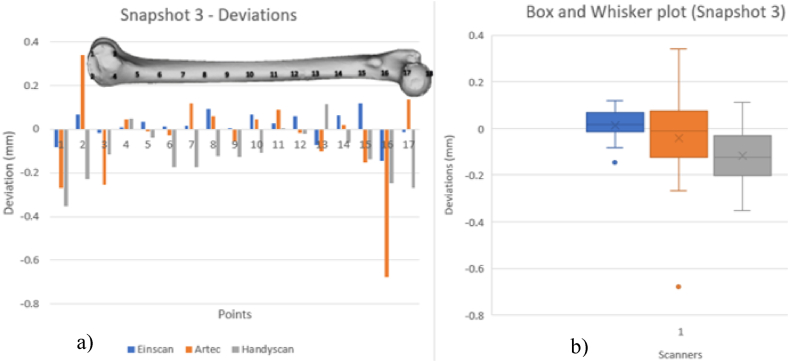
Deviations of scanners (left) and Box and whisker plot for Snapshot 3 (right).

**Fig. 23:**
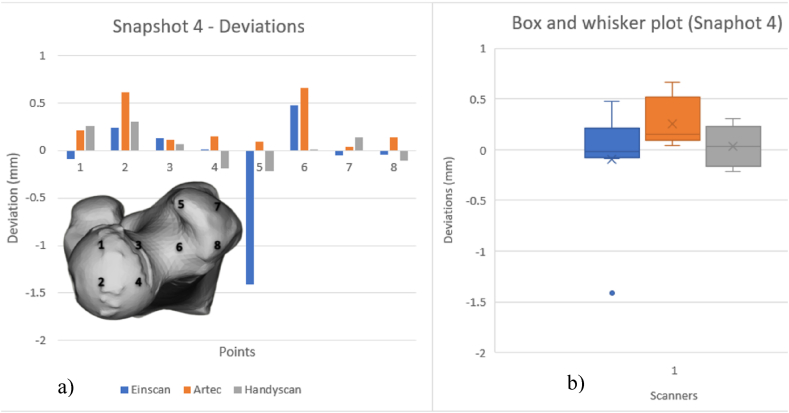
Deviations of scanners (a) and Box and whisker plot for Snapshot 4 (b).

Fig. 20, Fig. 21, Fig. 22, Fig. 23 show VX Inspect statistics on model deviations from the mesh file. Histograms display local deviation numbers, to compare scanner performance. The histograms provide one scanner's model's mm deviation from the others at various snapshot points. The analysis of these diagrams can be conducted by considering the various scanning technologies employed and the orientation of the scanned part. VX Inspect's toggle map function displayed the color map and deviations for each local reading. Reference planes that were identical to all of the models were difficult to come by because of the model's organic shape. Snapshots were nearly comparable, though, thus it was possible to compare regional model variations rather well. Appendices A, B, and C give the reports that were taken out during the analysis. See Appendices A, B, and C for the matching color maps for each of the snapshots from Fig. 20, Fig. 21, Fig. 22, Fig. 23.

In In the following discussion, the Artec EVA-M, Einscan HX, and Handyscan 700 will be designated as scanners Scanner 1, Scanner 2, and Scanner 3, respectively.

Fig. 20 shows longitudinal section deviations. Curvature and deviation are linear in the left histogram. The model's core has fewer deviations than its extremities.

Fig. 21 demonstrates that most scanner deviations are consistent and positive. The scanning orientation of different scanners affects points 5 and 8. For scanner 1 and scanner 2 the model was placed vertically, but for scanner 3 was tilted. Thus, scanners 1 and 2 deviate similarly to scanner 3. With a smaller interquartile range and a histogram showing a reduced deviation, scanner 3 was the better option in this perspective. This is because scanner 3 has a lower resolution and scanning orientation.

Fig. 22 displays a similarity of observation as on Fig. 20 along longitudinal deviations. The left histogram shows how curvature increases deviation. Middle-model deviations are smaller than those at the edges, supporting the observation. The scanners behave differently in Fig. 20, Fig. 22. Scanning results were better with scanners 1 and 2 compared to scanner 3.

Fig. 23 demonstrates consistent deviations for all three scanners except the outlier at point 5. At locations 5 and 6, all three scanners' maximum and minimum deviation values were found. Scanners 2 and 3 outperformed scanner 1 on the femur model.

#### 4.2.3 MeshLab results

**Fig. 24:**
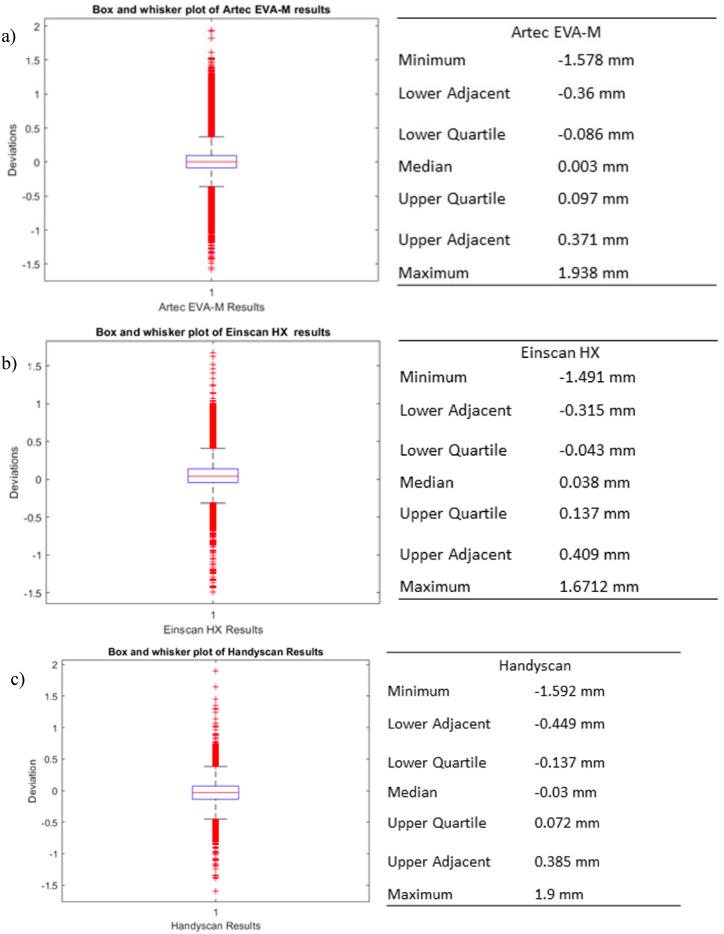
Plots of each scanner's deviation data using box and whisker (a) Artec Eva-M, (b) Einscan HX, and (c) Handyscan.

The box and whisker plots for the distance measured from the reference are shown in Fig. 24. The data of deviation shows extreme values are outliers. Since there are positive and negative values, comparing the mean value is unrealistic. RMS values will be compared. The original STL CAD file is this analysis's reference model. Therefore, these results incorporate AM process variations. Since outliers are not considered, this study shows scanning process deviance well. Findings are regularly distributed because all plots are symmetric. Since the data follows a normal distribution, the discrepancy lies between the two extremes of the box plots. Table 6 shows the range of deviation.

**Table 6:** Summary of the deviation range and RMS of the 3D scanners.

	Range of Deviations (mm)	RMS (mm)	Range (mm)
**Artec EVA-M**	−0.36 to 0.371	0.26	0.731
**Einscan HX**	−0.315 to 0.409	0.206	0.724
**Handyscan 700**	−0.449 to 0.385	0.181	0.834

The deviations for both structured light scanners are pretty much the same. The Handyscan 700, on the other hand, had differences averaging about 0.1 mm higher. The scanning deviation's RMS value is about 0.22 mm on average, and the scanners' average errors are between −0.375 mm and 0.388 mm.

**Fig. 25:**
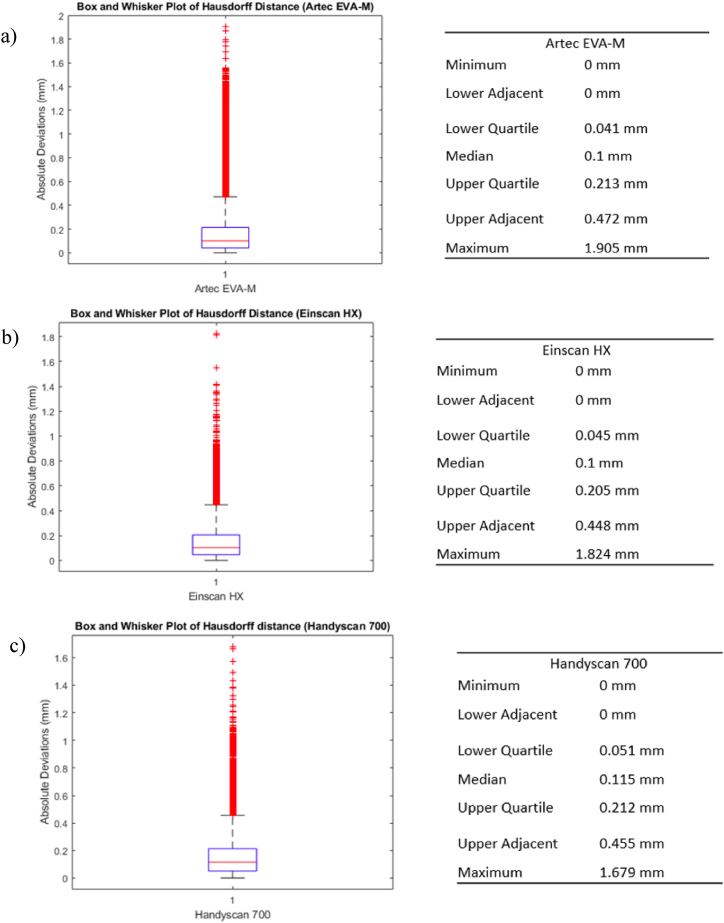
Hausdorff distance diagrams using box and whisker (a) Artec Eva-M, (b) Einscan HX, and (c) Handyscan.

After aligning the models, Hausdorff distance filter was applied in the MeshLab. The box and whisker graphs depicted in Fig. 25 were subsequently generated in Matlab using the data extracted from MeshLab. The median value of each value is around 0.1 mm. Table 7 indicates 3D scanner interquartile ranges.

**Table 7:** Each 3D scanner's Hausdorff distance interquartile ranges.

	Interquartile Ranges of Hausdorff Distance (mm)	Adjacent Minimum - Maximum Values Range (mm)	Mean (mm)
Artec EVA-M	0.041–0.213	0–0.472	0.174
Einscan HX	0.045–0.205	0–0.448	0.151
Handyscan 700	0.051–0.212	0–0.455	0.154

The 3D-printed model deviates 0.46 mm from the STL on average. Interlaboratory research into tolerance femur model reconstruction by Soodmand et al. [35] recommended medical model reconstruction within 1 mm. Considering this factor, the findings indicate that the 3D printed model derived from the reconstruction of medical images falls within acceptable limits of tolerance.

## 5 Conclusion

RE was most used in medicine for solid modeling, additive manufacturing, virtual surgical planning, quality control, and generative design. In addition to its direct and indirect uses in healthcare, RE offers practical alternatives to traditional methods of digital model development.

The process involved segmenting, reconstructing, and 3D printing a medical image on an industrial FDM printer. This technique has demonstrated the ability to alter medical images. Solid modeling and analysis of comparative deviations were accomplished by reverse engineering the printed model. The experimental investigation showed the convenience, efficiency, and advantage of non-contact scanning. RE is efficient with non-contact scanning.

The current research shows that medical model dimensional correctness may be evaluated using reverse engineering techniques; the deviations measured vary from −0.375 mm to 0.388 mm, with a range of around 0.763 mm and an average root-mean-square (RMS) of 0.22 mm. The outcomes show that the 3D printed model's mean deviation ranges from approximately 0.46 mm, with a mean value of approximately 0.16 mm. Reverse engineering proves it is a vital tool for quality control and dimensional assessment. Ultimately, when employing the suggested approach, do variance analysis on numerous samples, utilizing various additive manufacturing technologies, in order to generate a substantial dataset. Utilize artificial intelligence (AI) and machine learning (ML) algorithms to include the data and determine the variation of the 3D printed object based on characteristics such as AM technique, material, slicing height, and other variables.

## CRediT authorship contribution statement

**Yosef Wakjira:** Writing – original draft, Supervision, Methodology, Formal analysis, Data curation, Conceptualization. **Navaneethan S. Kurukkal:** Methodology, Investigation, Data curation, Conceptualization. **Hirpa G. Lemu:** Writing – review & editing, Visualization, Supervision, Software, Resources, Conceptualization.

## Declaration of competing interest

The authors declare that they have no known competing financial interests or personal relationships that could have appeared to influence the work reported in this paper.
