# Supplementary material for: Assessment of the accuracy of 3D printed medical models through reverse engineering
Source: Heliyon. 2024 May 24;10(11):e31829. doi: 10.1016/j.heliyon.2024.e31829 (PMC11153247; doi:10.1016/j.heliyon.2024.e31829)
Supplement: Multimedia component 1 [file mmc1.docx]

1. **Einscan femur models**

**Snapshot 1**


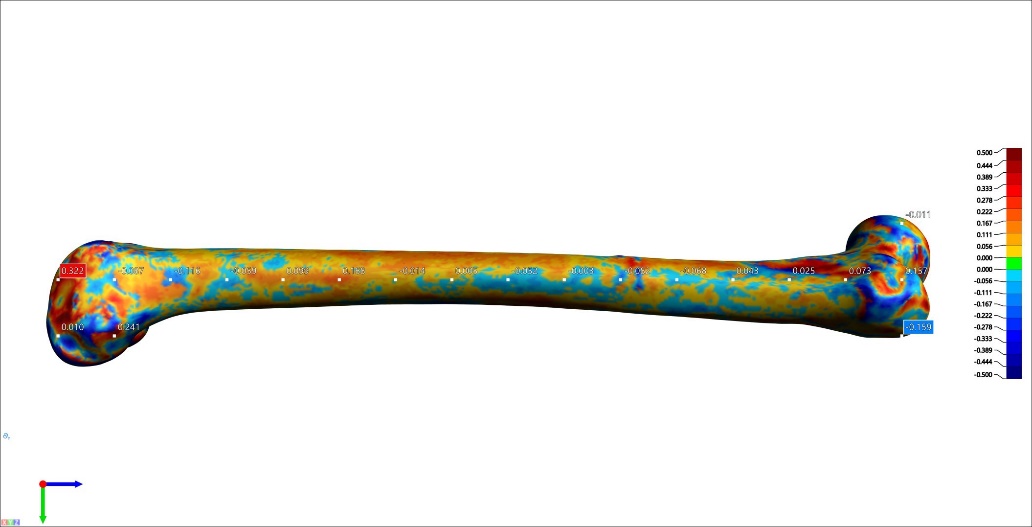


**Snapshot 2**


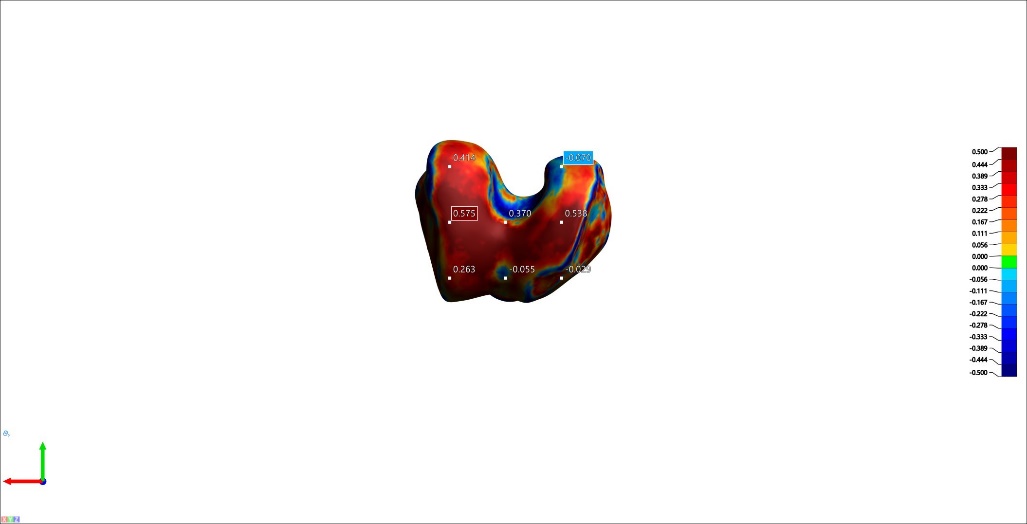


**Snapshot 3**


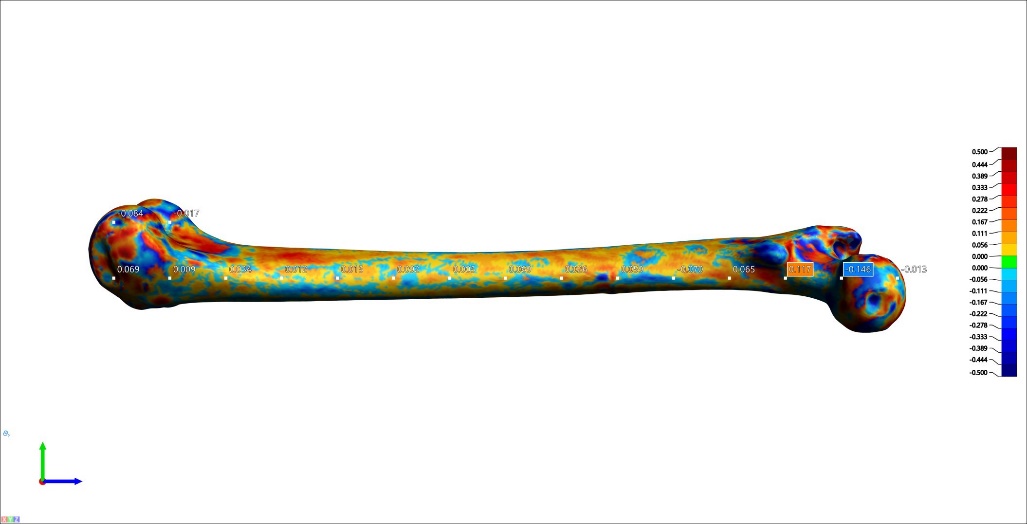


**Snapshot 4**


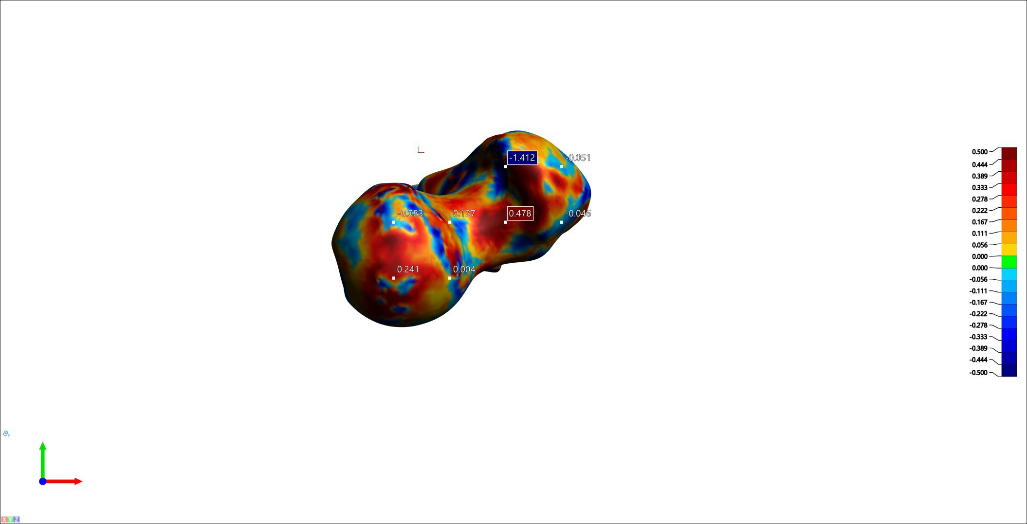


**Snapshot 5**


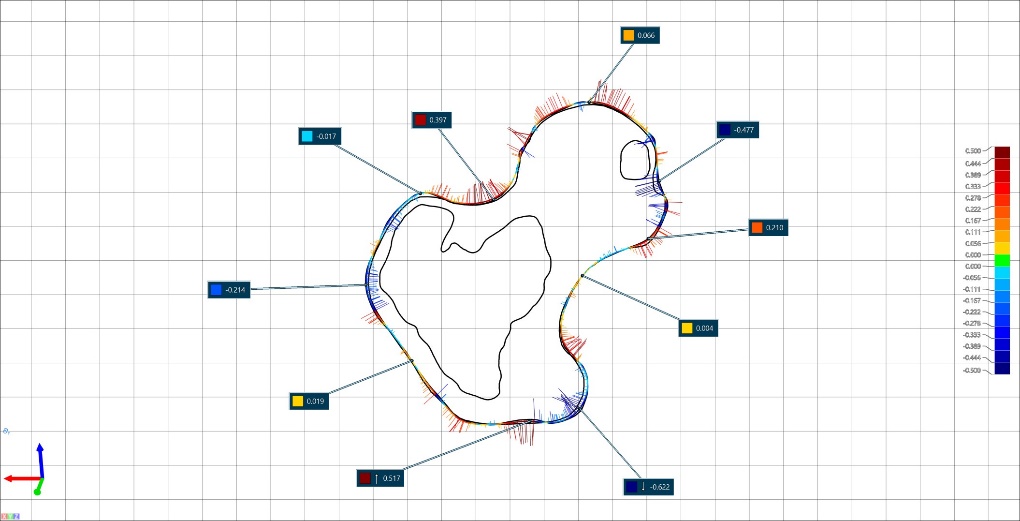


**Snapshot 6**


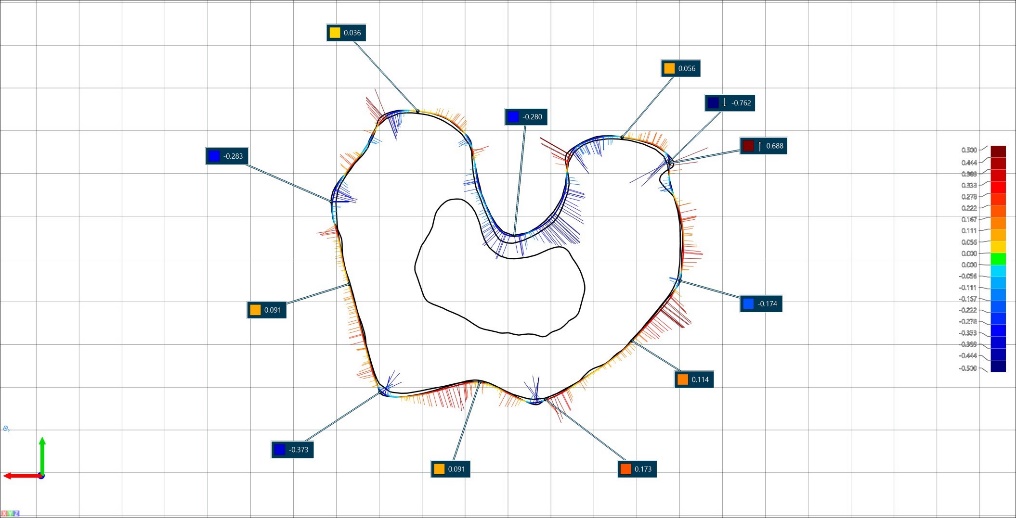


**Snapshot 7**


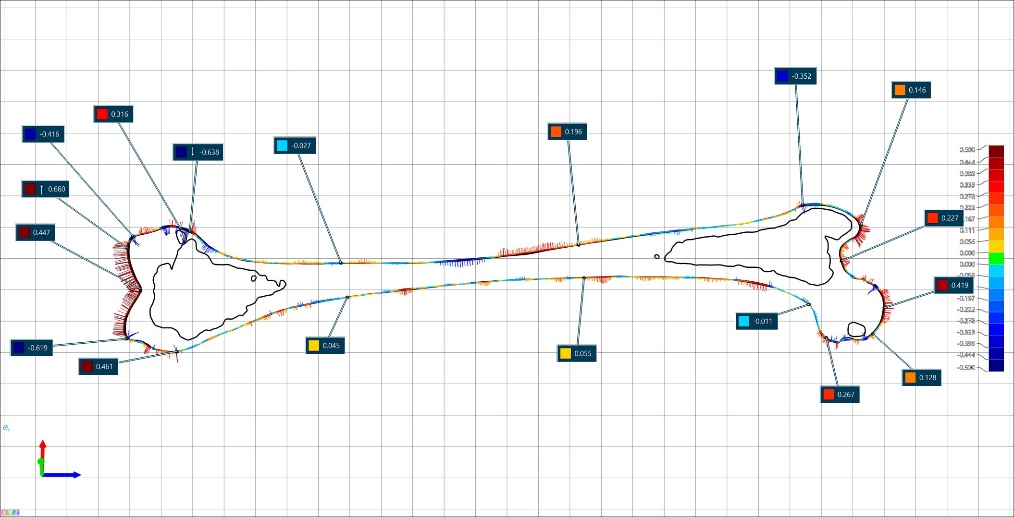


1. **Handyscan femur models**

**Snapshot 1**


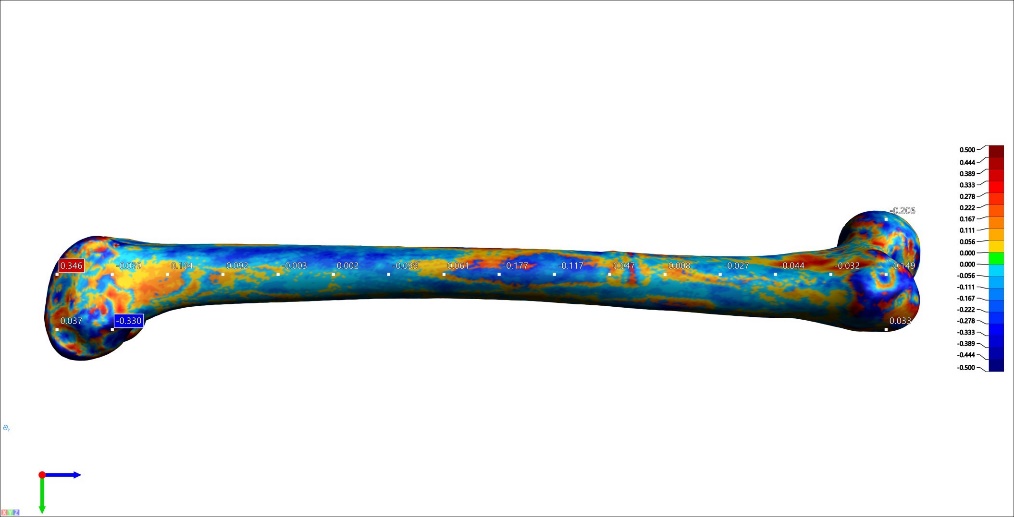


**Snapshot 2**


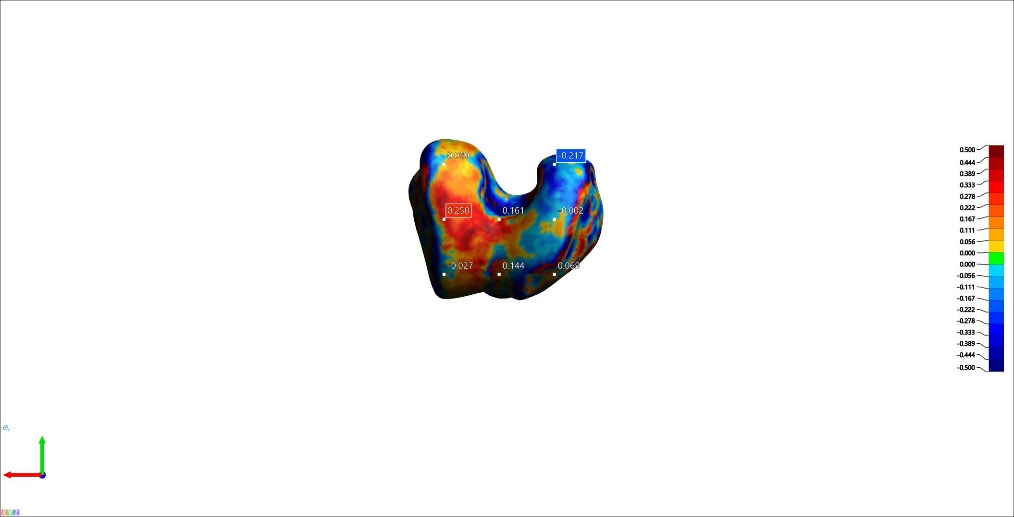


**Snapshot 3**

**
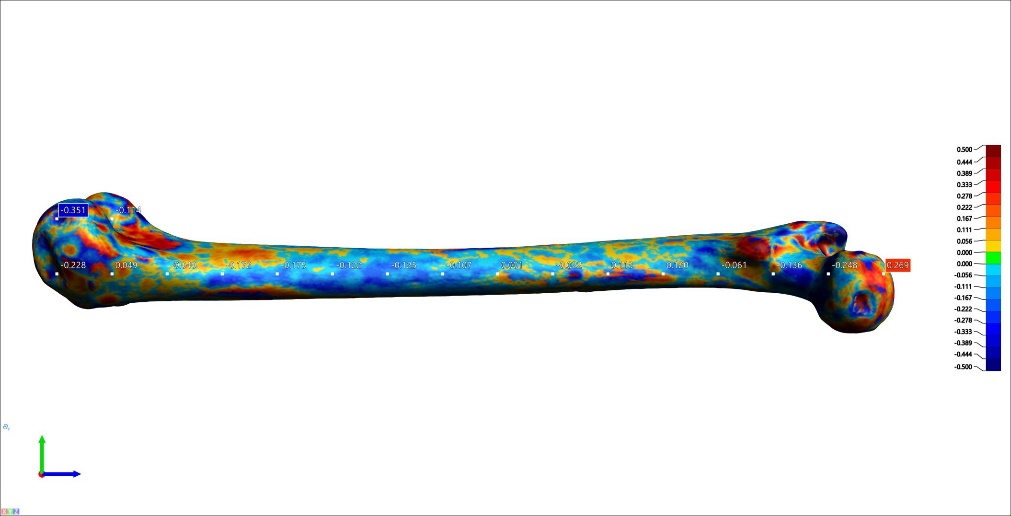
**

**Snapshot 4**


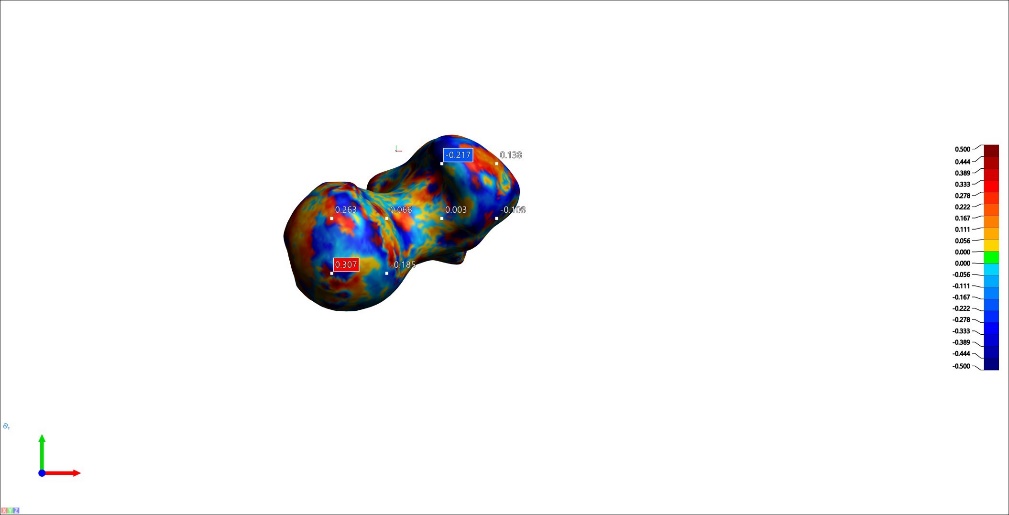


**Snapshot 5**


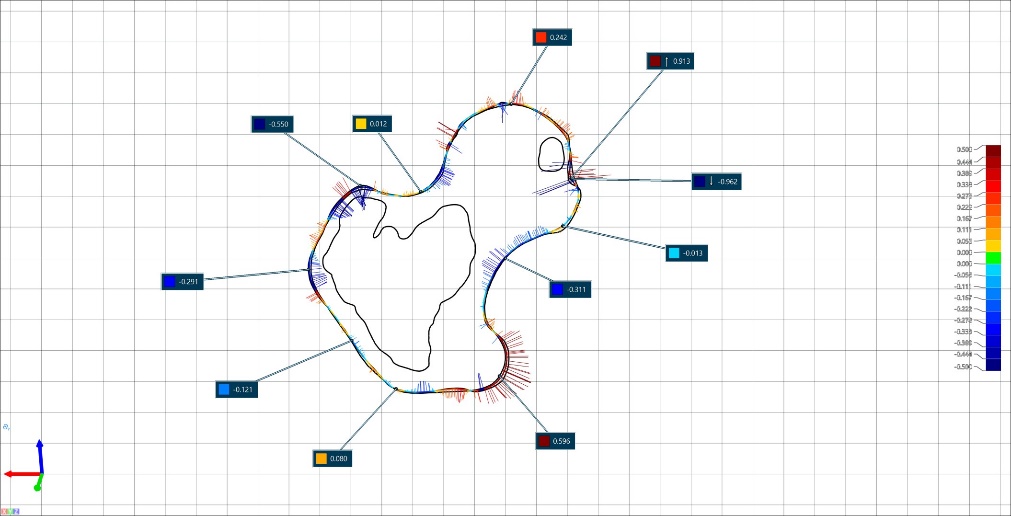


**Snapshot 6**


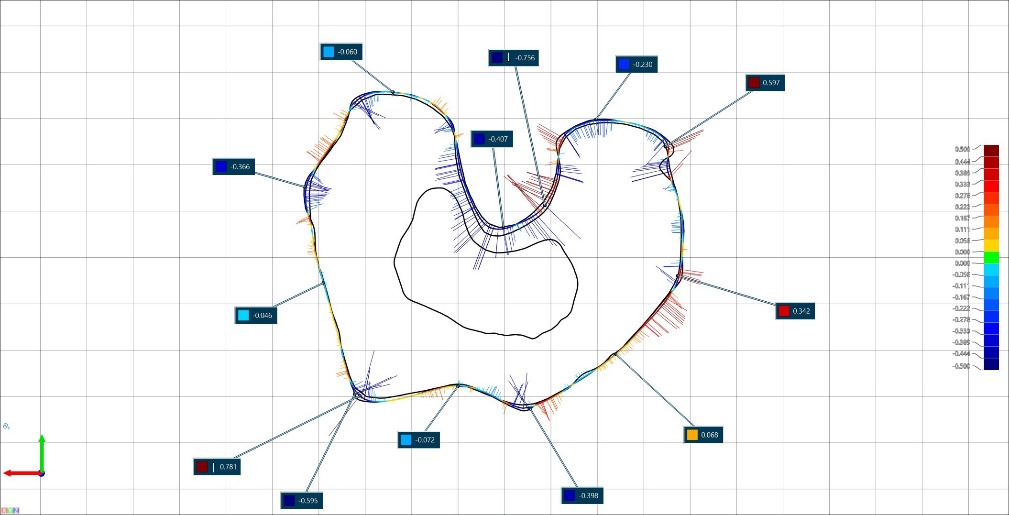


**Snapshot 7**

**
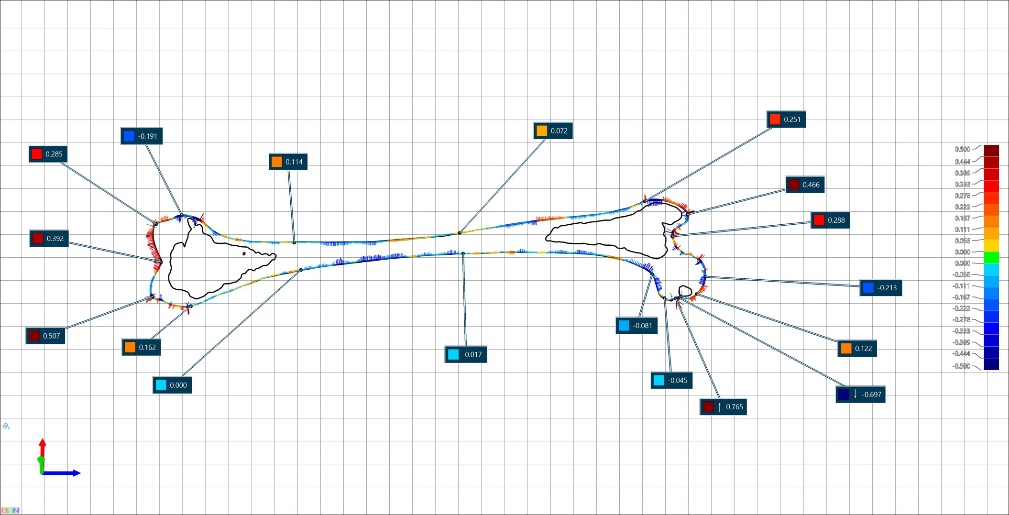
**

1. **Artec EVA-M femur models**

**Snapshot 1**

**
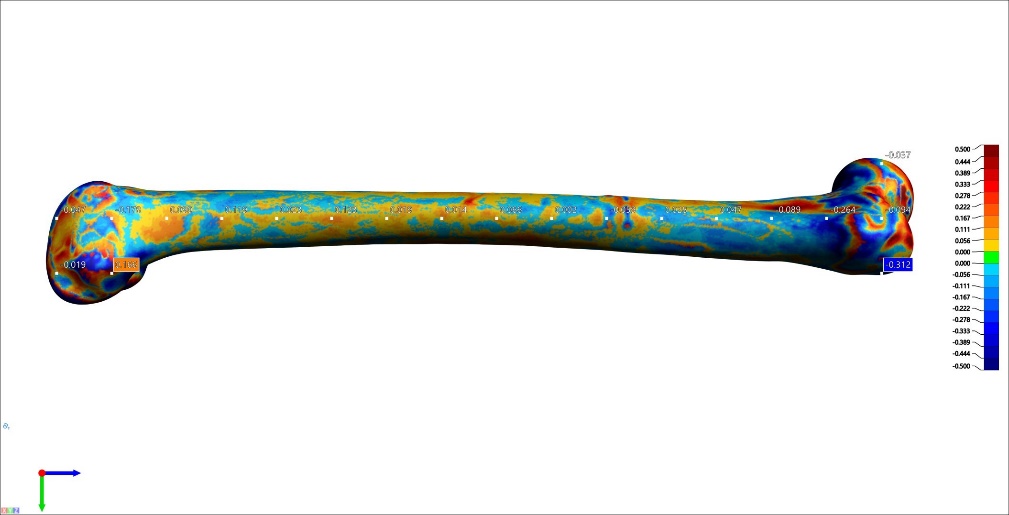
**

**Snapshot 2**

**
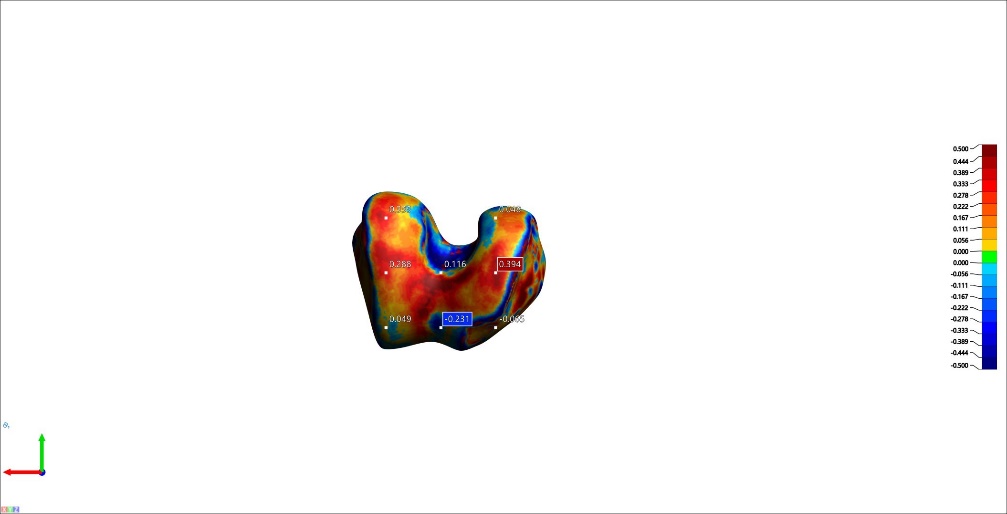
**

**Snapshot 3**

**
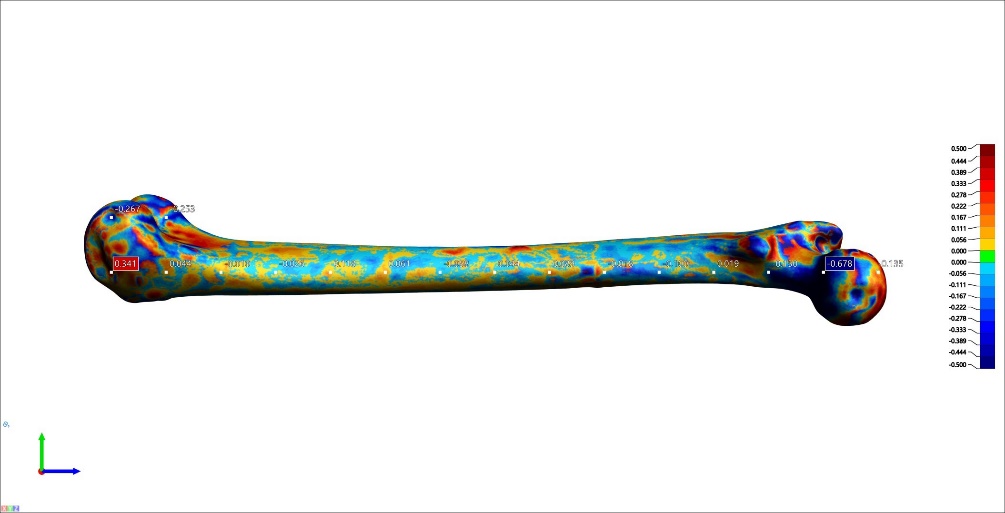
**

**Snapshot 4**

**
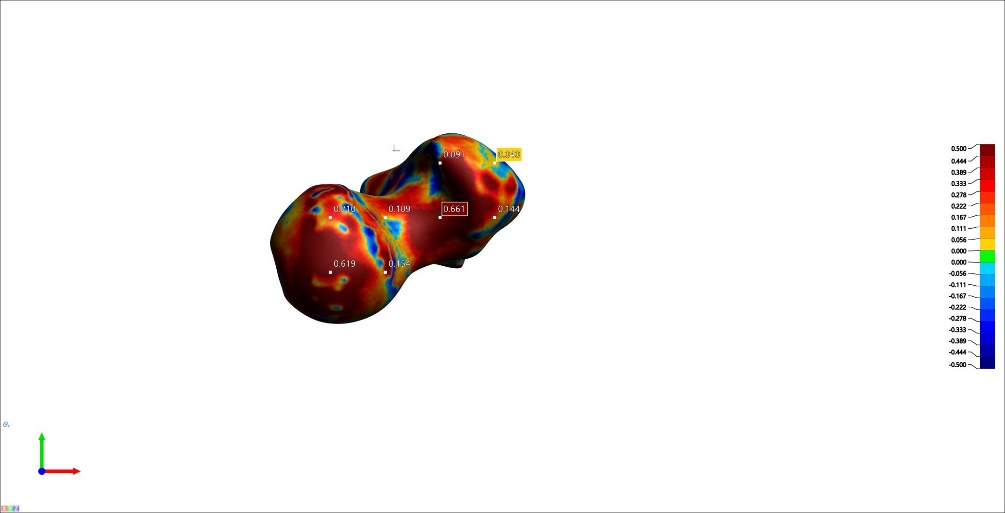
**

**Snapshot 5**

**
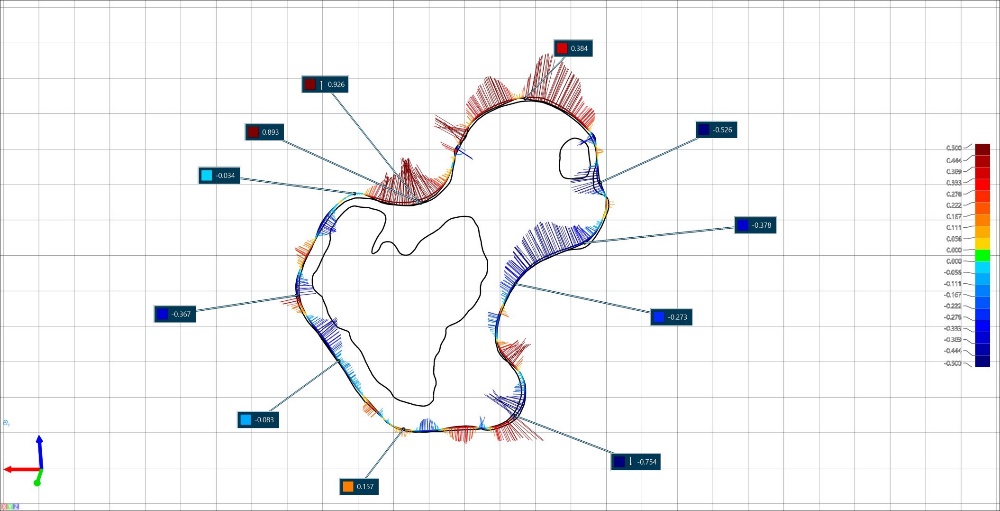
**

**Snapshot 6**

**
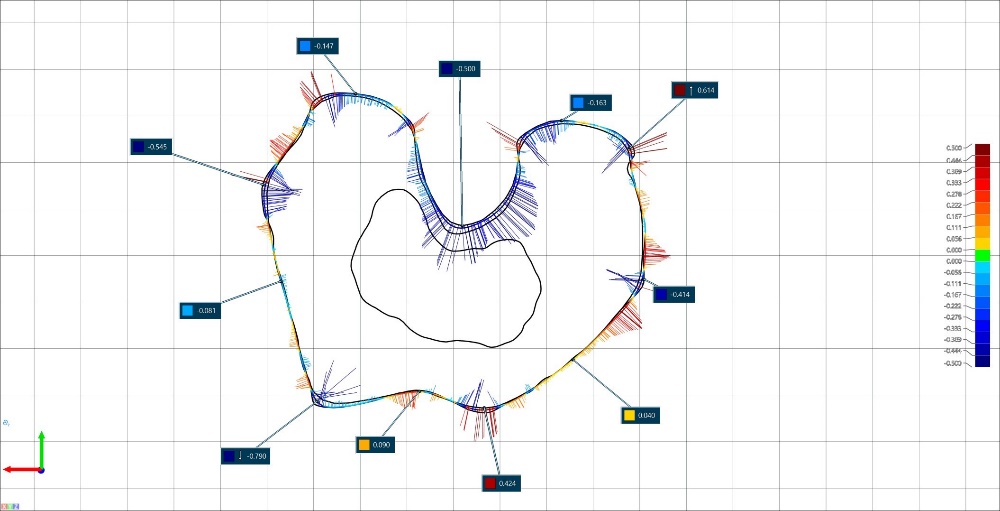
**

**Snapshot 7**

**
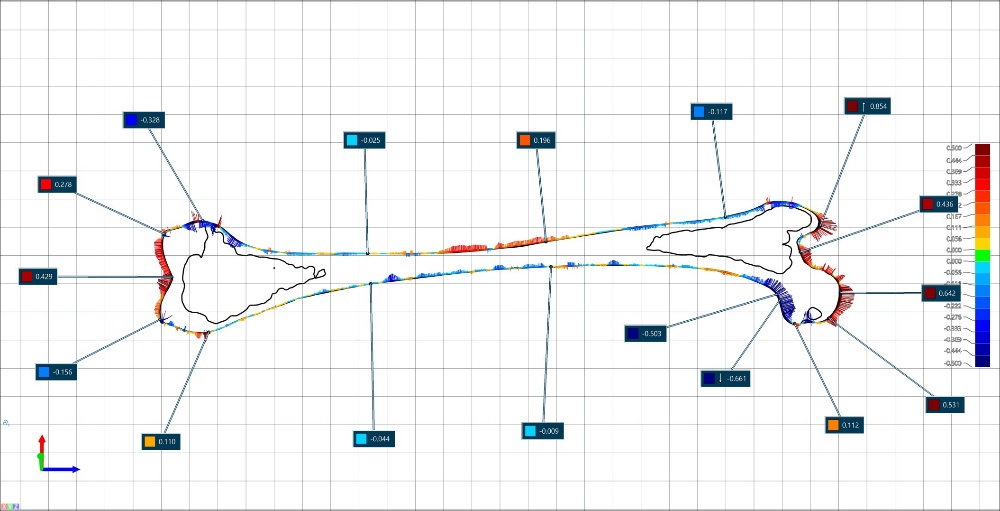
**
